# Supplementary material for: Facilitators and barriers for completion of the diagnostic process among people with presumed tuberculosis in Central Uganda
Source: PLOS Glob Public Health. 2025 Sep 19;5(9):e0004808. doi: 10.1371/journal.pgph.0004808 (PMC12449001; doi:10.1371/journal.pgph.0004808)
Supplement: S1 File — (DOCX) [file pgph.0004808.s001.docx]

**S1 File: The Consolidated Criteria for Reporting Qualitative Studies (COREQ) Checklist**

| **Domain 1: research team and reflexivity** | | |
| --- | --- | --- |
| **Personal characteristics** | | |
| 1. Interviewer/facilitator | Which author(s) conducted the interview or focus group? | All interviews and focus group discussions were conducted by two trained qualitative researchers (RN and VK), *lines 225, pg 10*. |
| 2. Credentials | What were the researcher’s credentials? (e.g. PhD, MD) | RN holds a master’s degree in Public Health and VK, maters in Sociology, *lines 227-228, pg 10.* |
| 3. Occupation | What was their occupation at the time of the study? | RN was a Research Associate at the University and the study coordinator while VK was a qualitative researcher on the project |
| 4. Gender | Was the researcher male or female? | RN is female and VK, male, *line 227, pg 10.* |
| 5. Experience and training | What experience or training did the researcher have? | Both RN and VK had training in qualitative research methodologies at their masters degree, undertook additional training in short courses and had previous experience with qualitative interviewing and analysis. |
| **Relationship with participants** | | |
| 6. Relationship established | Was a relationship established prior to study commencement? | There was no known prior relationship between the researchers and participants. |
| 7. Participant knowledge of the interviewer | What did the participants know about the researcher? (e.g. personal goals, reasons for doing the research) | During the consent and introduction process, participants were informed where the researchers worked and the purpose of the research. |
| 8. Interviewer characteristics | What characteristics were reported about the interviewer/facilitator? (e.g. bias, assumptions, reasons and interests in the research topic) | Biases arising from previous work experience in TB clinics were reported in lines 854-856, pg 35. |
| **Domain 2: study design** | | |
| **Theoretical framework** | | |
| 9. Methodological orientation and theory | What methodological orientation was stated to underpin the study? (e.g. grounded theory, discourse analysis, ethnography, phenomenology, content analysis) | The methodological orientation underpinning the study was phenomenology where we explored facilitators and barriers for completion of the TB diagnostic process. The socio-ecological model by Bronfenbrenner was the theoretical underpinning.  This is indicated in lines 104, pg 5 and lines 256-258, pg 11. |
| **Participant selection** | | |
| 10. Sampling | How were participants selected? (e.g. purposive, convenience, consecutive, snowball) | All participants were selected purposively. Participants for the IDIs were selected based on their completion status of the TB diagnostic process (completed diagnostic process for TB/not completed at all). FGD participants was based on the time spent on treatment ie one month. Key informants were purposively selected based on their role in providing TB services at the health facility. That is either providing TB screening, health education, counselling, testing and providing treatment to those diagnosed.  Indicated in *lines 167-169, 190-192 and 215-217.* |
| 11. Method of approach | How were participants approached? (e.g. face to face, telephone, mail, e-mail) | Names of eligible participants for both the IDIs and FGDs sampled from the presumptive and TB unit treatment registers were handed over to the TB facility linkage officers who contacted the patients through phone calls using the contacts recorded in the registers at the health facilities. The TB facility linkage officers were introduced to the study and taken through the objectives and procedures for inviting participants for the interviews. During the phone conversation, they introduced the study to the potential participant as guided by the research team and invited the participant to come to the health facility for the face-to-face interview if willing to participate.  *Lines 204-212, pg 9.* |
| 12. Sample size | How many participants were in the study? | Twenty-five in-depth interview participants and 20 key informants. Forty-two took part in focus groups (n=6-8 participants).  *Lines 296-301, pg 12.* |
| 13. Non-participation | How many people refused to participate or dropped out? Reasons? | We invited over 32 participants for 1D1s, only 25 turned up for interviews and all those who turned up, consented to participate.  For FGDs, we invited 54 participants out of which 42 turned up for the discussions.  The information on why participants did not turn up to the health facilities for the interviews is limited as they had promised to come but never showed up. |
| **Setting** | | |
| 14. Setting of data collection | Where was the data collected? (e.g. home, clinic, workplace) | All interviews and FGDs were conducted at health care facilities where the participants had been presumed with TB.  *Lines, 208-210, pg 9 and lines 233-234, pg 10.* |
| 15. Presence of non-participants | Was anyone else present besides the participants and researchers? | Only the researchers were present. |
| 16. Description of sample | What are the important characteristics of the sample? (e.g. demographic data, date) | Out of 25 in-depth interview participants interviewed, 15 were female and almost a third aged between 45 years and above. Almost half (11/25) were farmers by occupation. We conducted six focus group discussions with 42 participants ranging between 6-8 per group, an average of seven participants. Most participants of the focus group (14), were aged 31-39 years of age and more than half (23), were females. Of the 20 key informants; three-quarters of them were female and most aged between 20-30 years (6) and 31-40 years (6).  *Lines 296-301, pg 12.* |
| **Data collection** | | |
| 17. Interview guide | Were questions, prompts, guides provided by the authors? Was it pilot tested? | Interview guides with probes were developed and used during the interviews and discussions. These were reviewed within the research team and approved by the research and ethics body.  *S2 File* |
| 18. Repeat interviews | Were repeat interviews carried out? If yes, how many? | No repeat interviews were carried out. |
| 19. Audio/visual recording | Did the research use audio or visual recording to collect the data? | All interviews and focus group sessions were audio-recorded.  *Lines 235-236, pg 10.* |
| 20. Field notes | Were field notes made during and/or after the interview or focus group? | Field notes were taken during the interviews and discussions which notes were sed during data analysis and interpretation.  *Lines 236-237, pg 10.* |
| 21. Duration | What was the duration of the interviews or focus group? | Focus groups lasted between 50 minutes and 1 hour while IDIs and KIIs lasted about 40-60 minutes.  *Line 189, pg 8 and line 203, pg 9* |
| 22. Data saturation | Was data saturation discussed? | Data saturation was not discussed. |
| 23. Transcripts returned | Were transcripts returned to participants for comment and/or correction? | Transcripts were not returned to participants. |
| **Domain 3: analysis and findings** | | |
| **Data analysis** | | |
| 24. Number of data coders | How many data coders coded the data? | Two researchers who conducted the interviews (RN and VK) independently coded the data.  *Lines 244-258, pgs 10-11* |
| 25. Description of the coding tree | Did authors provide a description of the coding tree? | A coding framework has been provided in *S1 File* |
| 26. Derivation of themes | Were themes identified in advance or derived from the data? | The themes were not pre-determined, they were derived from the data. From the findings, themes were determined based on the constructs of the socio-ecological model and researchers experiences during data collection.  This information is included in *lines 256-258, page 11* |
| 27. Software | What software, if applicable, was used to manage the data? | ATLAS.ti software (version 6).  *Line 256, pg 11.* |
| 28. Participant checking | Did participants provide feedback on the findings? | Findings from this study were later shared back to the patients during an insight workshop for their feedback and validation to which they agreed to them and went ahead to suggest intervention components to address these interventions in the next phase of this large study.  However, we did not ask participants to prioritise these factors.  This information is included in *lines 262-264, pg 11.* |
| **Reporting** | | |
| 29. Quotations presented | Were participant quotations presented to illustrate the themes/findings? Was each quotation identified? (e.g. participant number) | Quotations have been presented throughout *the results section for the different themes*, with unique identifiers. |
| 30. Data and findings consistent | Was there consistency between the data presented and the findings? | The data presented is a true reflection of the field findings. We have also endeavoured to report the study findings in a clear, consistent manner.  Yes, major themes are clearly presented in the *results section.*  Yes, there were minor themes not selected as indicated in the analysis framework in *S1 File.* These themes were either not commonly mentioned by participants or were related to a bigger theme and hence merged. |
| 31. Clarity of major themes | Were major themes clearly presented in the findings? |  |
| 32. Clarity of minor themes | Is there a description of diverse cases or discussion of minor themes? |  |
