# Supplementary material for: Facilitators and barriers for completion of the diagnostic process among people with presumed tuberculosis in Central Uganda
Source: PLOS Glob Public Health. 2025 Sep 19;5(9):e0004808. doi: 10.1371/journal.pgph.0004808 (PMC12449001; doi:10.1371/journal.pgph.0004808)
Supplement: S2 File — (DOCX) [file pgph.0004808.s002.docx]

**S2 File: Interview guides**

**In-depth interview guide- Formative phase**

*(****Respondents:*** *People with presumed TB who either finished or did not finish the screening process and Confirmed patients with TB who either initiated treatment within 1 month or did not initiate treatment)*

**Participant socio-demographic characteristics**

| **Variable** | **Response** |
| --- | --- |
| **Date of Interview** |  |
| **Interview ID**  *(IDI_01_MIT_RN)*  *Where IDI- Interview type; 01- Number of interview: MIT- First three letters of the facility, Mityana; RN- Initials of the Research Assistant* |  |
| **Name of Research Assistant** |  |
| **Name of Health facility** |  |
| **Age in complete years** |  |
| **Sex** |  |
| **Religion** |  |
| **Occupation** |  |
| **Area of Stay** |  |

1. **Beginning question**
2. What do you know about the disease TB? (***Probe for: how common it is in the respondent’s community, How it spreads and How it can be prevented.)***
3. What could be the importance of testing for TB? What problems may arise if one does not test for TB when advised to?
4. **Pre-diagnosis loss to follow-up**

3. Please tell me a story of your journey from the time you were presumed to have TB up to when you received your results. (***Probe for: Reactions/ feelings when informed of suspected TB, how they proceeded to test, counselling received, how they obtained their results etc)***

***If participant completed the screening process, ask Qn 3. If participant did not complete screening process, ask Qn 6***

*4.* I understand that when you were suspected to have TB, you went ahead and submitted the samples for testing/ conducted other investigations required and also received your results. What could have enabled you to;

I) Submit the sample required or do other investigations required? ***(Probe for individual, social, community and health system facilitators [pay attention to what happens at the points of sample collection, and submission of sputum])***

ii) Receive your results? ***(Probe for individual, social, community and health system facilitators [pay attention to what happens at the point of receipt of results])***

iii) Please tell me what the results of your investigations were. Positive or negative?

5. There are some presumed people with TB who don’t submit the samples or receive back their results. From your experience as you went through the process what do you think hinders those patients from:

I) submitting the samples? ***(Probe for individual, social, community and health system factors [cost involved, time to visit the health facility, distance to the facility, availability of treatment services, time taken to receive the services, and support from health workers])***

ii) Receiving back their results? ***(Probe for individual, social, community and health system factors [cost involved, time to visit the health facility, distance to the facility, availability of treatment services, time taken to receive the services, and support from health workers])***

6. What do you think can be done to ensure that all people presumed with TB submit samples, or carryout the required investigations and also come back to receive their results**? *(Probe for what can be done at an individual level, family level, societal level, health facility level and policy level)***

**For those who did not complete the screening process;**

7. I understand that you were presumed to have TB but unfortunately did not finish the screening process. At what point in this diagnosis process did you stop? ***(Probe for I) submit the required sample or do other required investigations for testing and or ii) receive back their results)***

8. Please share with me what could have happened that led you not to **(ask in reference to the answer given in 6 above)**

I) Submit the sample required or do other required investigations ***(Probe for individual, social [social/peer support/stigma influences], community, environmental and health system facilitators [cost of testing, time to test, distance to the facility, availability of the TB testing services, time taken to receive the services, and support from health workers])***

ii) Receive your results? ***(Probe for individual, social [social/peer support/stigma influences], community, environmental and health system facilitators [cost of testing, time to test, distance to the facility, availability of the TB testing services, time taken to receive the services, and support from health workers])***

9. There are some people with presumed TB who manage to submit the samples and receive back their results. From your experience as you went through the diagnostic process what do you think enables those patients to:

i) Submit the samples or do other required investigations? **(Probe *for individual, social, community and health system factors [pay attention to what happens at the points of sample collection, submission of sputum and receipt of results])***

ii) Receiving back their results? **(Probe *for individual, social, community and health system factors [pay attention to what happens at the points of sample collection, submission of sputum and receipt of results])***

10. What do you think can be done to ensure that all people with presumed TB submit samples, do the required tests and also come back and collect their results? ***Note:*** *Ensure you re-echo to the participant what they mentioned as barriers and facilitators before and ask for solutions for each.* ***(Probe for what can be done at an individual level, family level, societal level, health facility level and policy level)***

***For presumptive TB who turned positive, proceed to Section C. If the patient was negative, skip to section D***

**C) Pre-treatment loss to follow-up**

11. Please tell me a story of your journey from the time you received the positive TB results *(****Probe: patient’s reactions/ feelings, understanding of TB disease, the need to start treatment, decision to start treatment, complication of not starting treatment in time)***

12. What could be the importance of starting treatment on time if found to have TB? What problems may arise if one does not start treatment if found to have TB?

13. Please tell us the treatment options you know or have heard of.

14. Please share with me if you started treatment when you were given TB positive results. If Yes, where did you initiate treatment from?

If No, proceed to QN 15-17

**If participant initiated treatment, ask Qns 12-14. If participant did not initiate treatment, ask Qns 15-17**

15. So you told me you went ahead and started treatment for TB after being confirmed. Did you start the treatment immediately? Yes/NO

a) If Yes, what could have enabled you to start the treatment immediately? ***(Probe for individual, social, community, environmental and health system facilitators)***

b) If No or there were delays in starting the treatment, what were the reasons for the delay or not starting treatment? ***(Probe for individual, social, community, environmental and health system facilitators)***

16. There are some confirmed people with TB who don’t start the treatment in time when informed of their status. From your experience as you went through the process what do you think hinders those patients from starting the treatment immediately? ***(Probe for individual, social [social/peer support/stigma influences], community and health system factors [costs involved, time to go to the facility, distance to the facility, availability of the TB treatment services, time taken to receive the services, and support from health workers])***

17. What do you think can be done to ensure that all confirmed people with TB start treatment immediately? ***(Probe for what can be done at an individual level, family level, societal level, health facility level and policy level)***

**For those who did not initiate treatment when they were confirmed to have TB**

18. You told me that you did not initiate treatment after you had been confirmed to have TB. What could have hindered you from starting the treatment immediately? ***(Probe for individual, social [social/peer support/stigma influences], community and health system facilitators [cost of testing, time to visit the health facility, distance to the facility, availability of treatment services, time taken to receive the services, and support from health workers])***

19. There are some confirmed TB patients who start the treatment immediately when informed of their status. From your experience as you went through the process what do you think enables those patients to start the treatment immediately? ***(Probe for individual, social, community and health system factors])***

20. What do you think can be done to ensure that all confirmed people with TB start treatment? ***(Probe for what can be done at an individual level, family level, societal level, health facility level and policy level)***

**D) Views on the intervention (SMS and MM incentive)**

**Preamble:** Given the challenges of people with presumed TB accessing care and the consequences of patients with TB not testing and also not starting on treatment, in this study we are proposing to send Short message services (SMS) to presumptive TB patients encouraging them to complete the diagnosis process. We will also give patients some money in form of transport to motivate those who have completed the TB diagnosis process and received their results, but also those who test positive for TB and start on the TB treatment. Short message services (SMS)-based interventions have been found to successfully improve adherence to TB treatment and ART in Uganda and elsewhere and uptake of other health interventions like immunization in children. In addition, incentives have also been found to improve health-care outcomes. It is against this background of the effectiveness of the SMS and incentive interventions in previous studies that we seek to use the same to test their effectiveness in reducing loss to follow up in the pre-diagnosis and pre-treatment phase.

**D1. Views on using SMS reminders to enhance linkage to care of presumptive and confirmed TB patients**

1. a) What are your views about using SMS messages to help presumptive TB patients complete the TB screening/testing process? How exactly do you think the SMS will help the presumptive TB patients to complete diagnostic process, receive their results as well as start treatment for those who test positive? (***Probe for SMS as a reminder, as alerts of results when ready etc)***
2. In your view, how willing would presumptive and or confirmed TB patients be to receive SMS encouraging them to;
3. Complete their TB screening/testing process. Please explain?
4. Receive their results. Please explain?
5. Starting treatment when positive? Please explain?
6. What problems are likely to be faced by presumptive or confirmed patients who receive SMS reminders encouraging them to complete the TB screening process as well as starting TB treatment? (***Probe for stigma, domestic violence, bleach of privacy, involuntary disclosure)***
7. What are your views on the design of the SMS reminder? Please explain your answer (one way or two way).
8. How should the message read or what words can we use in the message to remind the patient. Please explain your answer.
9. What languages should be used in delivering the SMS messages?
10. How often should the messages be sent to the patients?
11. What time is most appropriate to send the messages to the patients? **(Morning, afternoon, night).**
12. What more do you think should be done if sending reminder SMS is to work better to encourage patients to complete diagnosis as well as starting TB treatment?

**D2. Views on using mobile money incentive to enhance linkage to care of presumptive and confirmed TB patients**

22 a) What are your views about the influence of mobile money incentive sent to presumptive and confirmed TB patients in helping them complete the screening/testing process? How exactly would the mobile money incentive help the presumptive and TB patient to complete the screening process or start treatment. Why do you think so?

b) How do you think the mobile money incentive would be interpreted by patients? *(****Probe for bribery, coercion, enabler etc).*** Why do you say that?

c) What problems are likely to arise from receiving money to help presumptive and TB patients to complete the TB screening process as well as starting TB treatment? (***Probe for misuse of the money incentive for other purposes, stigma, domestic violence, involuntary disclosure)***

d) What are some of the gender concerns that may raise about the mobile money incentive?

e) What are your views on the amount of the mobile money incentive that should be given? **Please explain.**

f) In your opinion, when do you think a patient should be given the mobile money incentive? i*.e EITHER after the patient has been identified as presumptive TB patient to help in the process of submitting sample or after submitting a sample specimen to help them come back for their results? OR after receiving their results or after initiating treatment for the pre-treatment phase.*

g) What are your views on the content of the message from the sender to the patient once the incentive has been sent to them? *(****Probe for branding of the sender and content. Why do you say so?)***

h) What more do you think should be done if sending mobile money incentive is to work better to encourage patients to complete diagnosis as well as starting TB treatment? *(****Probe for receiving the money as a refund and not before, what form-mobile money or cash, goods vs mobile money)***

i) What other strategies would you think of apart from reminder SMS and mobile money incentive that can be used to reduce loss to follow-up among presumptive and confirmed TB patients?

**E. Concluding question**

23. We have now come to the end of this discussion; I appreciate your time in responding to all the questions raised. Is there anything else you would want to share, probably something we have not talked about that would help us understand why some patients complete the diagnosis process as well as starting treatment while others do not?

**THANK YOU**

**Focus group discussion interview guide- Formative phase**

*(****Respondents:*** *Patients who were* ***presumed to have TB and TB*** *patients who have been on treatment for less than one month)*

**Interview identifiers and Participant socio-demographics**

| **Variable** | **Response** |
| --- | --- |
| **Date of Interview** |  |
| **Interview ID**  *(FGD_01_KAW_Male)*  *Where FGD- Interview type; 01- Number of interview: KAW- First three letters of the facility; Male- type of the participants* |  |
| **Names of facilitator and note taker** |  |
| **Name of Health facility** |  |
| **District of the interview** |  |

**Socio-demographics**

| **No** | **Name** | **Age** | **Sex** | **Occupation** | **Area of Stay** |
| --- | --- | --- | --- | --- | --- |
|  |  |  |  |  |  |
|  |  |  |  |  |  |
|  |  |  |  |  |  |
|  |  |  |  |  |  |
|  |  |  |  |  |  |
|  |  |  |  |  |  |
|  |  |  |  |  |  |
|  |  |  |  |  |  |
|  |  |  |  |  |  |

**Beginning question**

1. What do you know about the disease TB? ***Probe: How common is it a problem in this community? How does it spread? How can it be prevented?***

**Pre-diagnosis loss to follow-up**

1. For us who are seated here, we were all presumed to have TB at some point. So tell us what happens when a presumptive TB patient is identified at the health facility till when they receive their results?
2. What could be the importance of testing for TB? What problems may arise if one does not test for TB when advised to?
3. When we are identified as presumptive TB patients, sometimes we may be required to I) submit samples do some tests and also ii) come back and collect our results when ready but some patients we don’t do so. In your view, what hinders some of us from:
4. Submitting the required samples or doing other requested investigations? ***(Probe for individual, social [social/peer support/stigma influences], community, and health system factors -pay attention to what happens at the points of sample collection, submission of sputum and receipt of results [cost of testing, time to visit the health facility, distance to the facility, availability of treatment services, time taken to receive the services, and support from health workers])***
5. Coming back to collect results when ready? ***(Probe for individual, social [social/peer support/stigma influences], community, and health system factors -pay attention to what happens at the points of sample collection, submission of sputum and receipt of results [cost of testing, time to visit the health facility, distance to the facility, availability of treatment services, time taken to receive the services, and support from health workers])***
6. We have some patients who complete this whole process of submitting the sample or doing other required investigations and receiving back their results. What do you think enables them to comply? **(Probe *for individual, social, community, environmental and health system facilitators)***
7. What do you think can be done to ensure that all presumptive TB patients submit samples, do other required tests and also come back and collect theirresults? (**Note:** Ensure you re-echo to the participant what they mentioned as barriers before and ask for solutions for each. ***(Probe for what can be done at an individual level, family level, societal level, health facility level and policy level)***

***If the FGD is for those who did not turn positive for TB, skip to section E***

***For an FGD of those who tested positive for TB, proceed to questions in section D***

**D.** **Pre-treatment loss to follow-up**

*7.* For us who are seated here, we were all confirmed to have TB at some point. So tell us what happens when a patient is confirmed to have TB at the health facility?

8. What could be the importance of starting treatment on time if found to have TB? What problems may arise if one does not start treatment if found to have TB?

9. Please tell us the treatment options you know or have heard of.

10. When we are confirmed to have TB, we are required to start taking drugs to get cured but also to stop spreading the disease in the community but some patients don’t do so. In your view, what hinders some of us from starting treatment? ***(Probe for individual, social [social/peer support/stigma influences], community, and health system factors -pay attention to what happens at the points of sample collection, submission of sputum and receipt of results [cost involved, time to visit the health facility, distance to the facility, availability of treatment services, time taken to receive the services, and support from health workers])***

11. We have some patients who start taking drugs once confirmed positive for TB. What do you think enables them to start? ***(Probe for individual, social, community, environmental and health system facilitators)***

12*.* What do you think can be done to ensure that all confirmed TB patients start taking drugs? (**Note:** Ensure you re-echo to the participant what they mentioned as barriers before and ask for solutions for each. ***(Probe for what can be done at an individual level, family level, societal level, health facility level and policy level)***

**E. Views on the intervention (SMS and MM incentive)**

**Preamble:** Given the challenges of presumptive TB patient accessing care and the consequences of patients with TB not testing and also not starting on treatment, in this study we are proposing to send Short message services (SMS) to presumptive TB patients encouraging them to complete the diagnosis process. We will also give patients some money in form of transport to motivate those who have completed the TB diagnosis process and received their results, but also those who test positive for TB and start on the TB treatment. Short message services (SMS)-based interventions have been found to successfully improve adherence to TB treatment and ART in Uganda and elsewhere and uptake of other health interventions like immunization in children. In addition, incentives have also been found to improve health-care outcomes. It is against this background of the effectiveness of the SMS and incentive interventions in previous studies that we seek to use the same to test their effectiveness in reducing loss to follow up in the pre-diagnosis and pre-treatment phase.

**E1. Views on using SMS reminders to enhance linkage to care of presumptive and confirmed TB patients**

1. a) What are your views about using SMS messages to help presumptive TB patients complete the TB screening/testing process? How exactly do you think the SMS will help the presumptive TB patients to complete diagnostic process, receive their results as well as start treatment for those who test positive? (***Probe for SMS as a reminder, as alerts of results when ready etc)***
2. In your view, how willing would presumptive and or confirmed TB patients be to receive SMS encouraging them to;
3. Complete their TB screening/testing process. Please explain?
4. Receive their results. Please explain?
5. Starting treatment when positive? Please explain?
6. What problems are likely to be faced by presumptive or confirmed patients who receive SMS reminders encouraging them to complete the TB screening process as well as starting TB treatment? (***Probe for stigma, domestic violence, bleach of privacy, involuntary disclosure)***
7. What are your views on the design of the SMS reminder? Please explain your answer (one way or two way).
8. How should the message read or what words can we use in the message to remind the patient. Please explain your answer.
9. What languages should be used in delivering the SMS messages?
10. How often should the messages be sent to the patients?
11. What time is most appropriate to send the messages to the patients? **(Morning, afternoon, night).**
12. What more do you think should be done if sending reminder SMS is to work better to encourage patients to complete diagnosis as well as starting TB treatment?

**E2. Views on using phone calls to enhance linkage to care of presumptive and confirmed TB patients**

1. a) What are your views about using phone calls to help presumptive TB patients complete the TB screening/testing process? How exactly do you think the phone calls will help the presumptive TB patients to complete diagnostic process, receive their results as well as start treatment for those who test positive? (***Probe for SMS as a reminder, as alerts of results when ready etc)***
2. In your view, how willing would presumptive and or confirmed TB patients be to receive phone calls encouraging them to;
3. Complete their TB screening/testing process. Please explain?
4. Receive their results. Please explain?
5. Starting treatment when positive? Please explain?
6. What problems are likely to be faced by presumptive or confirmed patients who receive phone calls encouraging them to complete the TB screening process as well as starting TB treatment? (***Probe for stigma, domestic violence, bleach of privacy, involuntary disclosure)***
7. What information should be delivered in the phone call to the presumptive TB patients? Please explain your answer.
8. What languages should be used when calling the presumptive TB patients?
9. How often should the phone calls be made to the patients?
10. What time is most appropriate to call to the patients? **(Morning, afternoon, night).**
11. What more do you think should be done if using phone calls is to work better to encourage patients to complete diagnosis as well as starting TB treatment?

**E3. Views on using both SMS reminders and phone calls to enhance linkage to care of presumptive and confirmed TB patients**

1. a) What are your views about using a combination of SMS reminders and phone calls to help presumptive TB patients complete the TB screening/testing process? How exactly do you think combined use of SMS and phone calls will help the presumptive TB patients to complete diagnostic process, receive their results as well as start treatment for those who test positive? (***Probe for SMS or phone call as a reminder, as alerts of results when ready etc)***
2. In your view, how willing would presumptive and or confirmed TB patients be to receive a combination of SMS and phone calls encouraging them to;
3. Complete their TB screening/testing process. Please explain?
4. Receive their results. Please explain?
5. Starting treatment when positive? Please explain?
6. What problems are likely to be faced by presumptive or confirmed patients who receive a combination of SMS and phone calls encouraging them to complete the TB screening process as well as starting TB treatment? (***Probe for stigma, domestic violence, bleach of privacy, involuntary disclosure)***
7. How should the combination of SMS and phone calls be delivered? Which one should be used first and why?
8. What should be the time between using either of the two *eg* what would be the time between sending an SMS and making a phone call?
9. How often should the SMS be sent and phone calls made to the patients?
10. What information should be delivered in the SMS and the phone call to the presumptive TB patients? Please explain your answer.
11. What languages should be used when sending the SMS and calling the presumptive TB patients?
12. What time is most appropriate to send sms reminders and call the patients? **(Morning, afternoon, night).**
13. What more do you think should be done if using a combination of SMS and phone calls is to work better to encourage patients to complete diagnosis as well as starting TB treatment?

**E4. Views on using mobile money incentive to enhance linkage to care of presumptive and confirmed TB patients**

16 a) What are your views about the influence of mobile money incentive sent to presumptive and confirmed TB patients in helping them complete the screening/testing process? How exactly would the mobile money incentive help the presumptive and Tb patient to complete the screening process or start treatment. Why do you think so?

b) How do you think the mobile money incentive would be interpreted by patients? *(****Probe for bribery, coercion, enabler etc).*** Why do you say that?

c) What problems are likely to arise from receiving money to help presumptive and TB patients to complete the TB screening process as well as starting TB treatment? (***Probe for misuse of the money incentive for other purposes, stigma, domestic violence, involuntary disclosure)***

d) What are some of the gender concerns that may raise about the mobile money incentive?

e) What are your views on the amount of the mobile money incentive that should be given? **Please explain.**

f) In your opinion, when do you think a patient should be given the mobile money incentive? i*.e EITHER after the patient has been identified as presumptive TB patient to help in the process of submitting sample or after submitting a sample specimen to help them come back for their results? OR after receiving their results or after initiating treatment for the pre-treatment phase.*

g) What are your views on the content of the message from the sender to the patient once the incentive has been sent to them? *(****Probe for branding of the sender and content. Why do you say so?)***

h) What more do you think should be done if sending mobile money incentive is to work better to encourage patients to complete diagnosis as well as starting TB treatment? *(****Probe for receiving the money as a refund and not before, what form-mobile money or cash, goods vs mobile money)***

i) What other strategies would you think of apart from reminder SMS and mobile money incentive that can be used to reduce loss to follow-up among presumptive and confirmed TB patients?

**F. Concluding question**

17. We have now come to the end of this discussion; I appreciate your time in responding to all the questions raised. Is there anything else you would want to share, probably something we have not talked about that would help us understand why some patients complete the diagnosis process as well as starting treatment while others do not?

**THANK YOU**

**Key Informant interview guide- Formative phase**

*(****Respondents:*** *In-charge and other key providers of out-patient department, TB clinic, ART Clinic and laboratory)*

1. **Participant socio-demographics**

| **Variable** | **Response** |
| --- | --- |
| **Date of Interview** |  |
| **Interview ID**  *(KII_01_KAW_RN)*  *Where KII- Interview type; 01- Number of interview: KAW- First three letters of the facility; RN- Initials of the Research Assistant* |  |
| **Name of Research Assistant** |  |
| **Name of Health facility** |  |
| **Name of facility department where the health worker executes their main duties** |  |
| **Age of health worker** |  |
| **Sex of health worker** |  |
| **Cadre of health worker** |  |
| **Years in health service** |  |

1. **Beginning question**

1. In your own view, what do you think of TB occurrence in this health facility? (***Probe for patient and health worker understanding of the disease severity and fatality.)***

1. **Pre-diagnosis loss to follow-up**

2. Please take me through the screening process for the presumptive TB patients at this health facility. ***(Probe for more information on triage/use of intensified case finding form/ tool; documentation; sample collection; referral for other investigation; and how results are received back by the patients).***

1. In some health facilities in Uganda and elsewhere, a certain percentage of presumptive TB patients get lost to follow up before they are diagnosed with TB. How much of a problem do you think this is here at this facility?
2. We understand there are patients who are presumed to have TB but do not complete the screening process.
3. In your view, what enables/ facilitates those who complete the screening process? ***(Probe for individual, social, community, environmental and health system facilitators [pay attention to what happens at the points of sample collection, submission of sputum and receipt of results])***

b) In your view, what hinders others from completing the screening process? ***(Probe for individual, social, community, environmental and health system factors [pay attention to what happens at the points of sample collection, submission of sputum and receipt of results])***

1. What do you think can be done to address these challenges/barriers you mentioned to help presumptive TB patients finish the screening/testing process? *(****Note:*** *Ensure you re-echo to the participant what they mentioned as barriers before and ask for solutions for each.)*
2. Are there categories of presumptive TB patients at this health facility who are likely to get lost to follow up more than others before they test for TB? Yes/ No
3. Who could those be? ***(Probe: Patients with no telephones, patients who come from far, age of the patient, drug users, etc). Why do you think they are more likely to get lost to follow-up compared to others?***
4. Are there differences between men and women when it comes to cases of loss to follow up among presumptive TB patients? In what ways? And what are some of the reasons why a certain gender is likely to get lost to follow up more than the other?
5. Are there differences between presumptive TB patients living with HIV and the HIV negative patients when it comes to cases of loss to follow up? Who is most likely to get lost to follow up and why?
6. **Pre-treatment loss to follow-up**
7. Tell me more about the presumptive TB patients whose results turn out positive for TB. How do they proceed to start treatment? ***(Probe for time taken to start TB treatment)?***
8. In some health facilities, some TB patients get lost to follow up before they are started on treatment. How common is this problem in this facility?
9. We understand there are patients who test positive for TB but do not start the TB treatment.
10. In your view, what makes it easy for patients to start treatment after being confirmed with TB? ***(Probe for individual, social, community, environmental and health system facilitators [pay attention to communication, support group, counselling, incentives etc])***
11. In your view, what hinders others from starting the TB treatment once they have tested positive? ***(Probe for individual, social, community, environmental and health system factors [pay attention to communication, support group, counselling, incentives etc])***
12. What do you think can be done to address these challenges/barriers that you mentioned to help confirmed TB patients to start the treatment? *(****Note:*** *Ensure you re-echo to the participant what they mentioned as barriers before and ask for solutions for each.)*
13. Are there categories of confirmed TB patients at this health facility who are likely to get lost to follow up more than others before they start treatment? Yes/ No
14. Who could those be? ***(Probe: Patients with no telephones, patients who come from far, age of the patient, drug users, etc). Why do you think they are more likely to get lost to follow-up compared to others?***
15. Are there differences between men and women when it comes to cases of loss to follow among confirmed TB patients? In what ways? And what are some of the reasons why a certain gender is likely to get lost to follow up more than the other?
16. Are there differences between confirmed TB patients living with HIV and the HIV negative patients when it comes to cases of loss to follow up before starting treatment? Who is most likely to get lost to follow up and why?
17. **Views on the intervention (SMS and MM incentive)**

**Preamble:** Given the challenges of presumptive TB patient accessing care and the consequences of patients with TB not testing and also not starting on treatment, in this study we are proposing to send Short message services (SMS) to presumptive TB patients encouraging them to complete the diagnosis process. We will also give patients some money in form of transport to motivate those who have completed the TB diagnosis process and received their results, but also those who test positive for TB and start on the TB treatment. Short message services (SMS)-based interventions have been found to successfully improve adherence to TB treatment and ART in Uganda and elsewhere and uptake of other health interventions like immunisation in children. In addition, incentives have also been found to improve health-care outcomes. It is against this background of the effectiveness of the SMS and incentive interventions in previous studies that we seek to use the same to test their effectiveness in reducing loss to follow up in the pre-diagnosis and pre-treatment phase.

**E1. Views on using SMS reminders to enhance linkage to care of presumptive and confirmed TB patients**

12 a) What are your views about using SMS messages to help presumptive TB patients complete the TB screening/testing process? How exactly do you think the SMS will help the presumptive TB patients to complete diagnostic process, receive their results as well as start treatment for those who test positive? ***(Probe for SMS as a reminder, as alerts of results when ready etc)***

b) In your view, how willing would presumptive and or confirmed TB patients be to receive SMS encouraging them to;

i) Complete their TB screening/testing process. Please explain?

ii) Receive their results. Please explain?

iii) Starting treatment when positive? Please explain?

c) What problems are likely to be faced by presumptive or confirmed patients who receive SMS reminders encouraging them to complete the TB screening process as well as starting TB treatment? ***(Probe for stigma, domestic violence, bleach of privacy, involuntary disclosure)***

d) What are your views on the design of the SMS reminder? Please explain your answer (one way or two way).

e) How should the message read or what words can we use in the message to remind the patient. Please explain your answer.

f) What languages should be used in delivering the SMS messages?

g) How often should the messages be sent to the patients?

h) What time is most appropriate to send the messages to the patients? (Morning, afternoon, night).

i) What more do you think should be done if sending reminder SMS is to work better to encourage patients to complete diagnosis as well as starting TB treatment?

**E2. Views on using mobile money incentive to enhance linkage to care of presumptive and confirmed TB patients**

13 a) What are your views about the influence of mobile money incentive sent to presumptive and confirmed TB patients in helping them complete the screening/testing process? How exactly would the mobile money incentive help the presumptive and Tb patient to complete the screening process or start treatment. Why do you think so?

b) How do you think the mobile money incentive would be interpreted by patients? ***(Probe for bribery, coercion, enabler etc).*** Why do you say that?

c) What problems are likely to arise from receiving money to help presumptive and TB patients to complete the TB screening process as well as starting TB treatment? ***(Probe for misuse of the money incentive for other purposes, stigma, domestic violence, involuntary disclosure)***

d) What are some of the gender concerns that may raise about the mobile money incentive?

e) What are your views on the amount of the mobile money incentive that should be given? **Please explain.**

f) In your opinion, when do you think a patient should be given the mobile money incentive? i.e EITHER after the patient has been identified as presumptive TB patient to help in the process of submitting sample or after submitting a sample specimen to help them come back for their results? OR after receiving their results or after initiating treatment for the pre-treatment phase.

g) What are your views on the content of the message from the sender to the patient once the incentive has been sent to them? ***(Probe for branding of the sender and content. Why do you say so?)***

h) What more do you think should be done if sending mobile money incentive is to work better to encourage patients to complete diagnosis as well as starting TB treatment? ***(Probe for receiving the money as a refund and not before, what form-mobile money or cash, goods vs mobile money)***

i) What other strategies would you think of apart from reminder SMS and mobile money incentive that can be used to reduce loss to follow-up among presumptive and confirmed TB patients?

**F. Concluding question**

14. We have now come to the end of this discussion; I appreciate your time in responding to all the questions raised. Is there anything else you would want to share, probably something we have not talked about that would help us understand why some patients complete the diagnosis process as well as starting treatment while others do not?

**THANK YOU**
