# Supplementary material for: Facilitators and barriers for completion of the diagnostic process among people with presumed tuberculosis in Central Uganda
Source: PLOS Glob Public Health. 2025 Sep 19;5(9):e0004808. doi: 10.1371/journal.pgph.0004808 (PMC12449001; doi:10.1371/journal.pgph.0004808)
Supplement: S3 File — (DOCX) [file pgph.0004808.s003.docx]

**S3 File: Analysis framework for** **factors for completion of the TB diagnostic process among patients with presumed TB in Central Uganda**

| **Codes** | **Reviewed codes** | **Themes** | **Exemplars** |
| --- | --- | --- | --- |
| Pain | Persistent symptoms and desire to get better | Facilitators for completing TB diagnosis | *“I had lost a lot of weight; I was no longer the way I was before and I was feeling so much pain and wanted to ensure that thing (pain) goes away. I had to do everything health workers tell me to do”* |
| Persistent symptoms |  |  |  |
| Wanting to get better |  |  |  |
| Social support | Social support |  | “*I coughed so much and they had bought me all the medicine and it did not work so my mother told me to come and test for T.B .”* |
| Knowledge about TB | Prior knowledge on TB |  |  |
| Experience or history of TB |  |  |  |
| Health talks on TB |  |  |  |
| Caring health workers | Caring health workers |  | *“What motivated me to come back to the hospital was the care that I received from the health workers. The health workers counsel you well and also assure us that TB cures and getting TB is not the end of the world.”* |
| Availability of cough monitors and facility linkage officers |  |  |  |
| Support from the TB departments |  |  |  |
| Obtaining same day results | Obtaining same day results |  | *“If I was not told that I have TB on that same day and also given drugs, I do not think I would have come back to hospital”* |
| Phone call to collect results | Calling of patients to collect results |  |  |
| Lack of transport | Lack of transport to return to the health facility | Barriers for completing TB diagnosis | *“The distance and the transport fees made it difficult for me. If it were nearby, I would have returned even that very day…. But the distance is long and you have to first gather some money before you can be able to come to the facility*.” |
| Distance to health facility |  |  |  |
| Fear of being seen testing for TB | TB and HIV related stigma |  |  |
| Non-disclosure of the testing process |  |  |  |
| Fear of spreading the disease |  |  |  |
| Fear of positive TB results |  |  |  |
| Fear of HIV positive results |  |  |  |
| Delayed results | Long waiting time |  | “*There is a lot of waiting especially at the laboratory. Patients get tired and leave without their results”* |
| Fear of TB results and treatment | Fear of positive TB results and treatment |  | *“…they are afraid because if they are found to be positive with TB, they have to be taking the drugs. They are afraid of taking the drugs. They will choose to walk away.”* |
| Fear of potential pill burden-those with HIV |  |  |  |
| Poor knowledge | Poor knowledge |  |  |
| Belief that whoever has TB has HIV |  |  |  |
| Belief that TB doesn’t cure |  |  |  |
| Inability to produce sputum | inability to produce sputum |  | *“Some disappear because they could not get the sample after giving them the container so they decide to go.”* |
| Poor quality samples |  |  |  |
| High sample load in the laboratory | High sample load |  | *“We work on samples from lower facilities in a day with the Gene Xpert machine. In a day we can work on thirty (30) samples or more than that in a day throughout the district. This gene expert takes one hour to run, just running and when it comes to sample collection, we can give it like three (3) hours. You find that time for patients to wait is long. It is too hard for them to wait.”* |
| Long turnaround time | Long turnaround time |  |  |
| Unavailability of some tests | Stock out of testing supplies |  |  |
| Stock out of laboratory supplies |  |  |  |
| Unclear appointment for collection of results | Unclear appointment for collection of results |  | *“They did not call me and they also did not give me a date so that when the date reaches i go back. But they did not tell me so I didn't know. When they did not give me a return date or call me, I went silent…what scared me was frequenting the hospital and what people would say and think about it, especially my mother. Had they called me, i would have come back.”* |
| Limited resources for communication |  |  |  |
| Poor documentation of patient contacts and address | Inadequate patient contact details |  |  |
| Incorrect patient telephone contact |  |  |  |
| Poor health worker attitude | Poor health worker attitude |  |  |
