# Supplementary material for: Facilitators and barriers for completion of the diagnostic process among people with presumed tuberculosis in Central Uganda
Source: PLOS Glob Public Health. 2025 Sep 19;5(9):e0004808. doi: 10.1371/journal.pgph.0004808 (PMC12449001; doi:10.1371/journal.pgph.0004808)
Supplement: S4 File — (PDF) [file pgph.0004808.s004.pdf]

RISK MITIGATION PLAN FOR THE STUDY ON USING SHORT MESSAGE SERVICE REMINDERS AND MOBILE MONEY INCENTIVES TO ENHANCE LINKAGE TO CARE OF PRESUMPTIVE TUBERCULOSIS PATIENTS IN UGANDA: A RANDOMISED CONTROLLED TRIAL

No.	Activity	Anticipated health risks		Risk mitigation measures
		**Research participants**	**Research assistants**	
1	Conducting a cohort analysis		Documents used in the cohort analysis maybe contaminated given that they are used by many people when entering data	❖ Documents will be reviewed within the hospital and in a well-ventilated room. No documents shall be carried out<br>❖ The table where the review is being conducted will have a hand sanitiser to enable the research assistants sanitise at every opportunity when reviewing the documents<br>❖ The table where the review is being conducted will be sanitised every 30 minutes<br>❖ All research assistants conducting the review will be required to keep their face masks on during the review
2	Training of research assistants		Contact with coronavirus contaminated surfaces	❖ We shall regularly decontaminate surfaces in the training room with JIK solution every after 2 hours.

HIGHER DEGREES, RESEARCH AND ETHICS COMMITTEE
APPROVED ON
18 JAN 2021
P. O. BOX 7072, KAMPALA
MAKERERE UNIVERSITY SCHOOL OF PUBLIC HEALTH

			Risk of infection with COVID-19 while interacting with each other	❖ Trainers and research assistants will be expected to hand-sanitise before entering and while leaving a training room.<br>❖ Face masks shall be a MUST wear at all times during the training.<br>❖ Research assistants will be required to maintain a social distance of about 2 metres at all times during the training<br>❖ Research assistants will be trained on the epidemiology of COVID-19, its signs and symptoms and preventive measures.<br>❖ A hygiene specialist will be invited to the training room to enforce the standard operating procedures<br>❖ A ministry of health toll free number and a list of individuals to contact in case of any COVID-19 alerts will be given to research assistants.
			Risk of infection with corona virus in case of contact with contaminated stationary (paper	❖ All research assistants will be required to hand-sanitize before and after contact with any stationary

HIGHER DEGREES, RESEARCH AND ETHICS COMMITTEE
APPROVED ON
18 JAN 2021
P. O. BOX 7072, KAMPALA
MAKERERE UNIVERSITY SCHOOL OF PUBLIC HEALTH

			work and pens)	(paperwork and pens).<br>❖ If possible sharing of stationary shall be minimized. We will ensure we provide enough stationary.
3	Travelling of the study team to the study sites		Contact with coronavirus contaminated surfaces during travel	❖ We shall regularly decontaminate surfaces in the car with JIK solution especially the door handles and chairs<br>❖ A hand sanitizer shall be made available in the car for the study team to constantly sanitize along the way
			Risk of infection with COVID-19 while interacting with each other during travel	❖ We will carry half capacity of the people per car and keep the windows open as much as possible<br>❖ The study team travelling will be required to maintain a social distance of about 2 metres at all times<br>❖ Everyone will be expected to wear a face mask at all times<br>❖ Anyone who has flu-like symptoms will not be allowed to travel with the rest of the team until fully investigated or recovered.
4	Data collection process and	Risk of infection with	Risk of infection with COVID-	❖ All field activities will be conducted

HIGHER DEGREES, RESEARCH AND ETHICS COMMITTEE
APPROVED ON
18 JAN 2021
P. O. BOX 7072, KAMPALA
MAKERERE UNIVERSITY SCHOOL OF PUBLIC HEALTH

	implementation of interventions	COVID-19 in case of contact with an infected research assistant	19 in case of contact with an infected research participant	while following standard measures of infection prevention and control for COVID-19 issued by the ministry of health.<br>❖ All interviews will be conducted in areas with enough ventilation.<br>❖ Both research participant and interviewer to maintain a social distance of about 2 metres<br>❖ Both the research participants and the data collection team will be required to wash hands or hand-sanitize using alcohol-based handrub before interviews, during interviews in case they touch any surface and after.<br>❖ It will be mandatory for both the research assistant and participants to correctly wear face masks during the interviews<br>❖ The research assistants will inform the participants about the signs and symptoms of COVID-19 in a language they understand, using approved IEC materials from the

HIGHER DEGREES, RESEARCH AND ETHICS COMMITTEE
APPROVED ON
18 JAN 2021
P. O. BOX 7072, KAMPALA
MAKERERE UNIVERSITY SCHOOL OF PUBLIC HEALTH

				ministry of health.<br>❖ In case of any alerts, the research assistants will provide the participant with a ministry of health toll free number and a list of individuals to contact for emergency response.
		Risk of infection with corona virus in case of contact with contaminated stationary (paper work and pens)	Risk of infection with corona virus in case of contact with contaminated stationary (paper work and pens)<br>HIGHER DEGREES, RESEARCH AND ETHICS COMMITTEE APPROVED ON 18 JAN 2021 MAKERERE UNIVERSITY SCHOOL OF PUBLIC HEALTH P. O. BOX 7072, KAMPALA	❖ All research assistants and study participants will be required to hand-sanitize using an alcohol based hand rub or a sanitizer before and after contact with any stationary (paperwork and pens).<br>❖ Where possible, sharing of stationary shall be minimized. We will ensure we provide enough stationary.
