# Supplementary material for: Facilitators and barriers for completion of the diagnostic process among people with presumed tuberculosis in Central Uganda
Source: PLOS Glob Public Health. 2025 Sep 19;5(9):e0004808. doi: 10.1371/journal.pgph.0004808 (PMC12449001; doi:10.1371/journal.pgph.0004808)
Supplement: Attachment — Submitted filename: Response to Editors comments_Facilitators and barriers.pdf [file pgph.0004808.s005.pdf]

"If I was not told that I have TB on that same day and given drugs, I don't think I would have come back to the hospital" Facilitators and barriers for completion of the diagnostic process among patients with presumed tuberculosis in Central Uganda

Point-by-point response to Editor's comments

Dear Editor,

Thank you for reviewing and providing valuable feedback for our manuscript titled **"If I was not told that I have TB on that same day and given drugs, I don't think I would have come back to the hospital" Facilitators and barriers for completion of the diagnostic process among patients with presumed tuberculosis in Central Uganda**

**Comment**	**Response**
1. Please complete all items on the Human Participants Research Checklist that are relevant for your submission	Thank you for the comment.<br>The Human Participants Research Checklist has been completed and uploaded on the system
2. You have cited file "Supplementary file 1" in the manuscript. However, there are no corresponding files uploaded to the submission. Please ensure that all files are present.<br>-Please select 'Supporting Information' for supplementary information files.	Comment noted.<br>Supplementary file 1 has been attached in the system.
Notes for the Human Participants Research Checklist	
1. Did you obtain ethics approval for this study?	Yes, ethical approval for the study was obtained and the approval letter has been attached in the system
2. If you prospectively recruited human participants for the study – for example, you conducted a clinical trial, distributed questionnaires, or obtained tissues, data or samples for the purposes of this study, please report in the Methods:<br>i. the day, month and year of the start and end of the recruitment period for this study.<br>ii. whether participants provided informed consent, and if so, what type was obtained (for instance, written or verbal, and if verbal, how it was documented and witnessed). If your study included minors, state whether you obtained consent from parents or guardians. If the need for	We prospectively recruited human participants for the interviews.<br>-The day, month and year of data collection were, between 18th April and 7th June 2023 as has been indicated in the manuscript, *line 170, page 9.*<br><br>-Participants provided written informed consent as indicated in *lines 209-210, page 11.*

consent was waived by the ethics committee, please include this information.	
3. If you are reporting a retrospective study of medical records or archived samples, please report in the Methods section:<br>i. the day, month and year when the data were accessed for research purposes<br>ii. whether authors had access to information that could identify individual participants during or after data collection	Comment noted.<br>This is not applicable to our study as our study involved primary data collection.

Sincerely,

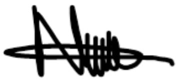

Rebecca Nuwematsiko
Corresponding author
