# Supplementary material for: Facilitators and barriers for completion of the diagnostic process among people with presumed tuberculosis in Central Uganda
Source: PLOS Glob Public Health. 2025 Sep 19;5(9):e0004808. doi: 10.1371/journal.pgph.0004808 (PMC12449001; doi:10.1371/journal.pgph.0004808)
Supplement: Attachment — Submitted filename: Response to editor comments_16th June 2025.pdf [file pgph.0004808.s006.pdf]

16th June, 2025

The Chief Editor,
PLOS Global Public Health

Dear Sir/ Madam

Re: Submission of revisions to the research manuscript titled *"Facilitators and barriers for completion of the diagnostic process among people with presumed tuberculosis in Central Uganda"*

On behalf of all co-authors, i hereby submit the response to comments for this manuscript titled *"Facilitators and barriers for completion of the diagnostic process among people with presumed tuberculosis in Central Uganda*."

We appreciate all the comments raised, they have helped to improve our manuscript, much appreciated.

Attached is the response to comments, tracked changes and clean copy.

Sincerely,

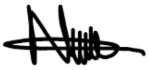

Ms. Rebecca Nuwematsiko, rnuwematsiko@musph.ac.ug
Department of Disease Control and Environmental Health
Makerere University School of Public Health

RESPONSE TO COMMENTS

**Comment**	**Response**
1. We are unable to access the Qualitative Data Repository using the link provided (https://data.qdr.syr.edu/dataset.xhtml?persistentId=doi%3A10.5064%2FF6BXWOWD&version=DRAFT) in order to confirm the availability of your minimal data for this study.<br><br>Before we can proceed with your submission, please confirm the public availability of your current Qualitative Data Repository link.<br><br>Alternatively, please upload the minimal anonymized data set necessary to replicate your study findings to a stable, public repository and provide us with the relevant URLs, DOIs, or accession numbers to access these data.	Thank you for the comment.<br><br>All the data and materials supporting this manuscript are within the paper and the supplementary file.<br>More data has now been uploaded in a different Data Repository *(Open Science Framework)* accessed at: https://osf.io/c2e5g/?view_only=859ba6ec709a4b538907f22c01a36dc6<br><br>This information has been updates in *lines 739-740, page 35.*
2. Please remove your figures from within your manuscript file, leaving only the individual TIFF/EPS image files. These will be automatically included in the reviewer's PDF. Please leave the figure captions in the manuscript.	Comment noted.<br>The figures were earlier on removed from the manuscript and only the caption was left in *line 261, page 13.*<br>Kindly advise if this is not the case on our side.
