# Supplementary material for: Facilitators and barriers for completion of the diagnostic process among people with presumed tuberculosis in Central Uganda
Source: PLOS Glob Public Health. 2025 Sep 19;5(9):e0004808. doi: 10.1371/journal.pgph.0004808 (PMC12449001; doi:10.1371/journal.pgph.0004808)
Supplement: Attachment — Submitted filename: Response to reviewers.pdf [file pgph.0004808.s007.pdf]

11th July, 2025

The Chief Editor,

PLOS Global Public Health

Dear Sir/ Madam

Re: Submission of revisions to the research manuscript titled *Facilitators and barriers for completion of the diagnostic process among people with presumed tuberculosis in Central Uganda*

On behalf of all co-authors, i hereby submit the response to comments for this manuscript titled *Facilitators and barriers for completion of the diagnostic process among people with presumed tuberculosis in Central Uganda*.

We appreciate the additional comments, they have helped to improve the manuscript, much appreciated.

Attached is the detailed response to comments, tracked changes and clean copy.

Sincerely,

Ms. Rebecca Nuwematsiko, rnuwematsiko@musph.ac.ug

Department of Disease Control and Environmental Health

Makerere University School of Public Health

Response to comments

Title: Facilitators and barriers for completion of the diagnostic process among people with presumed tuberculosis in Central Uganda

**Comment**	**Response**
**Journal Requirements**	
Please review your reference list to ensure that it is complete and correct. If you have cited papers that have been retracted, please include the rationale for doing so in the manuscript text, or remove these references and replace them with relevant current references. Any changes to the reference list should be mentioned in the rebuttal letter that accompanies your revised manuscript. If you need to cite a retracted article, indicate the article's retracted status in the References list and also include a citation and full reference for the retraction notice.	Thank you for the comment.<br>We have reviewed the reference list again and there are no retracted papers.
**Reviewers' comments**	
1. The term 'failed' or 'failing' to produce sputum is still used in a few instances, for example lines 423, 424, 426 and 427.	The comment has been noted.<br>The word failed or failing to produce sputum has been replaced with "unable" in the various sentences in *lines 415, 416, 417, 418, 419, 420, 438, 441 on pages 21-22 and line 577, page 28.*
2. Line 58: 'Pre-diagnosis and pretreatment loss of patients facilitates continued spread of TB in the community, poor treatment outcomes for those infected and death' – suggest re-wording 'those infected' for person/s or those with TB.	Thank you for the comment.<br>The sentence has been edited and the word "those infected" replaced with "persons with TB" in *line 56, page 4.*<br><br>*"Pre-diagnosis and pre-treatment loss of patients facilitates continued spread of TB in the community, poor treatment outcomes for persons with TB and death (4-8)."*
3. Line 99 in revised version: please review use of round bracket/parentheses after Mityana District) - there seems to be an extra closing parenthesis at the end.	Thank you for noting this.<br>This was a typing error and the parenthesis has been deleted in *line 95, page 6.*

4. Suggest mentioning that the audios were transcribed by the researchers who conducted the interviews – as per the comment/clarification provided in the review table.	Comment noted.<br>This information has been added in the manuscript for clarification as suggested in *lines 203-204, page 10.*<br><br>*"Audio recordings were transcribed verbatim in English by the researchers who conducted the interviews (VK and RN)."*
5.The closing sentence of the Conclusion mentions the TB & HIV same day testing policy. I would recommend making it clearer which policy this refers to (ie an existing policy - by study facilities or a ministerial policy etc), and what the stigma is associated with (with the policy, with HIV) so the closing sentence is clear and well defined.	Thank you for the comment.<br>The Uganda National HIV Testing Services Policy and Implementation Guidelines recommend that all people with active or those presumed for TB should be tested for HIV at the same time as indicated on pages 65-66 (https://library.health.go.ug/sites/default/files/resources/UGANDA%20HTS%20POLICY%20AND%20IMPLEMENTATION%20GUIDELINES%202016%20%282%29.pdf).<br><br>From participant views, the stigma was associated with being tested for HIV.<br><br>In light of this, the related concluding sentence has been revised to clarify the information above in *lines 748-751, page 36.*<br><br>*The national policy for same day testing of TB and HIV should be accompanied with heightened community awareness and counselling to minimize the associated stigma that comes with HIV testing which was not anticipated by the patients.*<br><br>The discussion has also been improved to include information on the TB/HIV testing policy and how it impacts TB patients in *lines 680-683, page 33.*<br><br>*This stigma was further complicated by the WHO and Uganda national guidelines on TB/HIV testing where patients are expected to test for TB and HIV at the same time which leads to stigma and fear especially if the HIV testing is offered in a facility that serves people living with HIV (MoH, 2016, WHO, 2012, Legido-Quigley et al., 2013).*
